# FACTORS AFFECTING BEHAVIORAL AND PSYCHOLOGICAL SYMPTOMS IN OLDER ADULTS WITH DEMENTIA

**DOI:** 10.1093/geroni/igz038.3293

**Published:** 2019-11-08

**Authors:** Hwang Sinwoo, Eunhee Cho

**Affiliations:** 1 Yonsei university, Seoul, Korea, Republic of; 2 Professor, College of Nursing, Yonsei University, Seoul, Korea, Republic of

## Abstract

The purpose of this study was to identify factors predicting behavioral and psychological symptoms of dementia(BPSD) in older adults with Dementia. This is a cross-sectional study, recruiting 157 participants from neurology general hospital as study subjects. Data collection was performed from June 2018 to May 2019. BPSD were classified using a modified version of the Cohen‐Mansfield agitation inventory(CMAI), which are physically non-aggressive behaviors(PNAB), physically aggressive behaviors(PGAB), verbally non-aggressive behaviors(VNAB), verbally aggressive behaviors(VAGB). The Cornell scale for depression in dementia(CSDD), korea activity of daily living scale(K-ADL), korean mini-mental state examination(K-MMSE), activity and sleep time through using actigraphy for 2weeks, salivary melatonin and cortisol level at 4 times a day done after waking up, after breakfast, before and after dinner, and medication were measured as influencing factors. The generalized linear mixed model analyses indicated that VNAB and VAGB were associated with severe depression(p<0.01, respectively), low melatonin level at the after waking up (p<0.05, respectively), and high melatonin level before dinner(p<0.05, respectively). On the PNAB and PGAB, severe depression(p<0.05, respectively) and high activity(p<0.05, respectively) showed great influences. These findings suggest that developing of intervention of BPSD must be started with detecting depression, ADL. Considering the factors of each type of symptom, tailoring an individual approach is recommended. In addition, this study identified that the activity through actigraphy and salivary melatonin measurement are useful tools to examine BPSD. It can be helpful in the objective evaluation of BPSD.

